# Assessing Good Prescription Writing Practice at the Pediatric Outpatient Clinic, Atbara Teaching Hospital, Sudan: A Clinical Audit

**DOI:** 10.7759/cureus.82334

**Published:** 2025-04-15

**Authors:** Razan Mohamed Elahdab Hassan, Rayan Samir Abdulhamed Hamimy, Ahmed Alshafei Elmahi, Huda Abdelfatah Ali, Wefag Yahya Adam Wadi

**Affiliations:** 1 Paediatrics and Child Health, Faculty of Medicine, University of Khartoum, Khartoum, SDN; 2 General Practice, Atbara Teaching Hospital, Atbara, SDN

**Keywords:** clinical audit, outpatient department, prescription audit, quality improvement projects, sudan, world health organization

## Abstract

Background

Inappropriate drug use constitutes a global health concern, especially in developing countries like Sudan, which exacerbates disease burden through medication errors, hence compromising patient safety and outcomes. Prescription auditing enhances clinical practice by improving prescription quality, aligning with WHO guidelines. This three-cycle clinical audit evaluated the impact of educational interventions on prescription writing practices, demonstrating their role in reducing prescription errors and optimizing healthcare quality, thereby addressing a pivotal public health challenge.

Methods

A prospective cross-sectional clinical audit was conducted in the Pediatric Outpatient Department of Atbara Teaching Hospital, Sudan. The audit spanned three cycles (August 2024 to February 2025), during which 60 randomly selected prescriptions per cycle were systematically evaluated. Compliance with WHO prescribing standards was assessed during the first cycle, after which an educational intervention was implemented before the second cycle. The results of this educational intervention were studied during both the second and third cycles to establish sustainability in improving prescription practices. Data analysis utilized Microsoft Excel (Microsoft Corporation, Redmond, Washington, United States) and IBM SPSS Statistics for Windows, Version 29.0 (Released 2023; IBM Corp., Armonk, United States), with categorical variables expressed as frequencies and percentages.

Results

The first cycle in this clinical audit on 60 pediatric outpatient prescriptions done in Atbara Teaching Hospital showed some fundamental defects; 86.7% (n=52) of the prescriptions did not contain patient name, 96.7% (n=58) did not state patient age, 85% (n=51) did not provide weight, and 100% (n=60) did not mention a diagnosis. Cycle three showed a significant improvement after this intervention, with 13.3% (n=8) of the prescriptions not containing the patient's name. Age documentation improved to 86.7% (n=52), weight documentation reached 91.7% (n=55), and diagnosis documentation improved to 96.7% (n=58). Inclusion of medication generic names increased from 50% (n=30) to 88.3% (n=53), drug strength documentation increased from 28.3% (n=17) to 90.0% (n=54), and the drugs were prescribed with dosage, frequency, route, and duration in the third cycle at the rate of 98.3% (n=59), 96.7% (n=58), 98.3% (n=59), and 86.7% (n=52), respectively. The documentation of the name of the prescriber increased from 3.3% (n=2) to 81.7% (n=49), whereas prescriptions including the prescriber's signature increased from 35% (n=21) to 93.3% (n=56). These interventions improved compliance with the WHO standards of prescriptions significantly.

Conclusion

This clinical audit demonstrates that integrating structured educational interventions with prescription practice reviews significantly enhanced the adherence to WHO prescribing standards in the Pediatric Outpatient Clinic at Atbara Teaching Hospital, thereby improving medication safety and patient outcomes.

## Introduction

The term prescription means an order from the prescriber for the dispensing or application of treatment as specified by the prescriber [1]. A prescription has a dual meaning: it signifies a pre-written instruction and implies an order from a prescribing authority. It is defined as "an order given by a prescriber to a dispenser to obtain a certain medicine or procedure to be applied". According to the World Health Organization (WHO), it is a therapeutic instruction given by the prescriber to the dispenser [2]. Prescribing is a critical clinical skill with far-reaching implications for patient safety, particularly in pediatric settings where doses require precise measurement and administration poses unique risks [3]. Prescribing errors are disproportionately common among clinicians in the early stages of practice [4]. Robust error prevention systems, training, and standardized protocols are essential to mitigate iatrogenic risks and improve therapeutic outcomes [5]. 

Medication errors occur frequently in pediatric care, with studies reporting a higher risk in children (31%) than in adults (13%) [5]. Incomplete or poorly written prescriptions remain a widespread issue across healthcare settings. For example, a study at Ribat University Hospital in Khartoum, Sudan, found that prescription forms routinely lacked critical data, including the patient’s full name (81.2%), prescriber details (93.3%), drug dose (59.7%), treatment duration (25.7%), and diagnosis (94%) [6]. 

In a descriptive cross-sectional study in three public healthcare centers in Khartoum, Sudan, which evaluated prescription quality among general practitioners and consultants, most prescriptions included basic patient and prescriber information, but essential details such as patient age, weight, drug strength, and usage instructions were frequently omitted [7]. Only 29.4% of the 504 analyzed prescriptions met completeness criteria. Similarly, an audit in Jizan, Saudi Arabia, revealed high rates of missing patient age (54.3%), weight (73.7%), refill instructions (97.8%), and therapy regimen (34.8%) [8]. These findings underscore the urgent need for interventions to reduce prescription-related errors. 

Despite existing studies on prescription errors in Sudan, there has been no systematic analysis of safe pediatric prescribing practices at Atbara Teaching Hospital benchmarked against international standards. This audit addresses this gap by evaluating compliance with WHO guidelines for prescription writing [9]. The lack of standardized local guidelines for comprehensive and safe prescriptions, coupled with the prevalent use of handwritten prescriptions in Sudanese governmental hospitals, highlights the necessity of clinical auditing. Furthermore, Sudan’s ongoing armed conflict exacerbates healthcare system challenges, emphasizing the need for audits to reinforce care quality. By identifying strengths and weaknesses in prescription practices, this audit provides actionable data to guide interventions, inform local guideline development, and enhance pediatric medication safety nationwide.

Aim, objectives, and standard

The aim and primary objective of this study was to evaluate prescription writing practices in the pediatric outpatient clinic at Atbara Teaching Hospital against WHO guidelines. The secondary objective of the study was to identify the common pattern of prescription writing errors in the pediatric outpatient clinic in order to develop interventions aimed at safe prescription practice.

The standards sought to be attained were adherence to WHO prescription guidelines: 80% of prescriptions should include patient details (name, age, weight), 80% of prescriptions should contain drug details (generic name, dose, frequency, route, and duration), and 80% of prescriptions should include prescriber details (name, signature, and date).

## Materials and methods

This prospective hospital-based clinical audit aimed to assess adherence to pediatric outpatient prescription guidelines at Atbara Teaching Hospital (August 2024-February 2025). It comprised three cycles: Cycle One (August 29-September 16, 2024), Cycle Two (October 17-November 4, 2024), and Cycle Three (December 5-31, 2024), evaluating compliance before/after an educational intervention using systematic prescription sampling. 

Audit area and population

This audit was conducted at the pediatric outpatient clinic of Atbara Teaching Hospital, a district hospital in Atbara, Nile River State. The Nile River State is a Northeastern state in Sudan, at the eastern bank of the Nile River, more specifically at the mouth of the seasonal Atbara River. Atbara is a commercial and agricultural center. The importance of Atbara city has increased following the armed conflict in Sudan as it became a destination for internally displaced persons (IDPs). As of February 21, 2024, Nile River State was reported to be hosting 792,234 IDPs from the start of the war in April 2023, which constitutes 9.9% of the total IDPs [10].

Atbara Teaching Hospital is a governmental hospital providing medical care in Atbara and its surrounding areas. Over time, the hospital has gradually become a training center in pediatrics for registrars, general practitioners, house officers, and students. The hospital consists of six working units with six consultants, residents, medical officers, and house officers.

The hospital has outpatient services and two inpatient wards, each with 15 beds. Additionally, Atbara Teaching Hospital operates a referral clinic that offers services four days a week, receiving approximately 40 patients per week. The hospital also houses a nutritional ward, a blood bank, and laboratory services with a high dependency unit (HDU), neonatal intensive care unit (NICU), and X-ray room.

Study population and inclusion and exclusion criteria

The study population consisted of all prescriptions written in the pediatric outpatient clinic at Atbara Teaching Hospital during the study period. Prescriptions issued for patients aged 0-18 years and for outpatients were included. Prescriptions for inpatients and for medications dispensed directly by the hospital (e.g., vaccines), incomplete prescriptions missing key information required for the audit, and prescriptions written on special forms (e.g., controlled medication prescriptions, refill medication prescriptions) were excluded.

Sampling and sample size

A total of 60 pediatric outpatient prescriptions were included per six-month audit period. Prescriptions were systematically selected via computer-generated random sampling (using a random-number generator) across all working days and clinical shifts to minimize bias. A blinded researcher performed the sampling, adhering to a protocol that included stratified randomization by clinical shift (morning/afternoon) and exclusion of incomplete prescriptions via a standardized checklist. This approach ensured statistical representativeness, feasibility, and transparency in the sampling methodology.

Data collection

Data were systematically collected using a WHO-aligned form in Google Forms (Google LLC, Mountain View, California, United States). This form was piloted on a small sample of prescriptions (n=5) to ensure clarity and ease of use. Any necessary revisions were made to the data collection form before full-scale data collection started. The following information were extracted from each prescription: Patient demographics (full name, age/ date of birth, weight), Prescription details (drug generic name, concentration, dosage form, dose, frequency, route of administration, duration), Prescriber details (name, signature ), Prescribing core indicators (number of medications per prescription, number of antibiotics per prescription and route of antibiotics per prescription), and Errors in prescription writing (legibility, use of abbreviations).

Statistical analysis

Data collected was subsequently imported into a Microsoft Excel spreadsheet (Microsoft Corporation, Redmond, Washington, United States). Data analysis was performed using IBM SPSS Statistics for Windows, Version 29.0 (Released 2023; IBM Corp., Armonk, United States) and Microsoft Excel. Descriptive statistics was used to summarize the characteristics of the prescriptions and to determine the frequency and percentage of adherence to each WHO standard.

Audit cycles

There were three cycles aimed at assessing prescription writing practice at the Pediatric Outpatient Clinic for further evaluation and improvement. 

First Cycle

The first cycle was conducted between August 2024 and September 2024, in which a total of 60 prescriptions were evaluated.

Intervention: Following the identification of deficiencies in prescription writing practices during the first audit cycle, an educational session was designed to introduce the WHO standard for good prescribing. The session consisted of a 30-minute interactive PowerPoint (Microsoft Corporation) presentation delivered by our team during the regular weekly pediatric department meeting. The presentation covered the first-cycle audit results, the WHO standard for good prescribing, the implications of improper prescriptions, and the objectives and target standards of the audit. This was followed by a group discussion on issues with current prescribing practices and a training session on achieving optimal prescription standards. A feedback form was distributed following the session. The session was attended by 50 participants, including consultants, pediatric registrars, medical officers, and house officers, as well as representatives from the quality improvement department and hospital pharmacy. To reinforce the training, educational posters were distributed throughout the outpatient department and shared on the hospital’s social media platforms.

Second Cycle

The second cycle was conducted between October 2024 and November 2024, following the implementation of interventions. Another 60 prescriptions were reviewed to evaluate improvement in practice. This cycle aimed to measure the impact of the interventions on compliance with prescription writing standards. Good prescribing practices were reinforced through regular reminders in the weekly departmental meetings and on the hospital’s social media platforms.

Third Cycle

The third cycle was conducted in December 2024, and 60 prescriptions were reviewed to evaluate improvement in practice. This cycle aimed to assess the sustainability of the improvement in the prescription writing practice following the intervention made prior to the second cycle. Following the third cycle, we presented the results of the three audits in the weekly meeting using a 15-minute PowerPoint presentation. The presentation emphasized the significance of the changes made and the importance of the sustainability of those changes. This was followed by a group discussion for suggested tools to achieve this sustainability.

Audit approval

This audit was approved by the Atbara Teaching Hospital, Ministry of Health, River Nile State (approval number: 60/B/2). There was no need for patients' informed consent for this audit.

Standards

The audit was conducted in alignment with the WHO guide to good prescribing, with modifications to reflect the characteristics of the local setting. Accordingly, a prescription should include: (i) Name, address, and telephone number of prescriber, (ii) Date, (iii) Generic name of the drug and strength, (iv) Dosage form, total amount, (v) Label: instructions, warning, (vi) Name, address, and age of patient, and (vi) Signature or initials of the prescriber.

Modifications to WHO standards for local adaptation

To further enhance the applicability of the WHO guidelines within the local settings of Sudan in general and the setting at Atbara Hospital specifically, certain modifications were made: (i) Inclusion of Patient's Weight: As we conducted the audit in a pediatric population, where most drug doses are weight-based, the patient's weight had been added as a required element on the prescription form. This allows for more accurate pediatric dosing. (ii) Exclusion of Prescriber Address and Phone Number: The prescriber's address and phone number were not included on the prescription form. Prescribers are typically identified in Sudan through their full name and place of work, which are deemed sufficient for identification and contact. Including the prescriber address and phone number information might reduce prescribers’ compliance due to the sensitivities in sharing this information in the local settings. This adaptation aimed to balance the principles of safe prescribing with the local norms and practical considerations.

## Results

In this audit, a total of 180 prescriptions were collected from the pediatric outpatient department of Atbara Teaching Hospital. The audit was done on the basis of prescription writing criteria developed by the WHO. The main objectives were to assess the level of prescribing practice compliance concerning completeness, accuracy, and conformity with given guidelines.

The collected prescriptions were compared to the WHO criteria based on four main categories: the prescription legibility, completeness of patient information including the diagnosis, completeness of drug information, and completeness of prescriber information, with each category containing more than one parameter.

Prescription legibility

Of the 60 first cycle prescriptions, 91.7% were legible, and this increased to 96.7% for both cycles two and three. An agreed definition to legibility was that a legible prescription needed to be clearly legible with no errors for four members of the audit team, a prescription failing to meet this was classified into either partially legible if three or two could clearly read it or not legible (illegible) if one or no one could read the prescription (Table 1).

**Table 1:** Legibility of prescriptions

First Cycle (n=60), n (%)			Second Cycle (n=60), n (%)			Third Cycle (n=60), n (%)		
Legible	Partially legible	Not Legible	Legible	Partially legible	Not Legible	Legible	Partially legible	Not Legible
55 (91.7%)	5 (8.3%)	0 (0%)	58 (96.7%)	2 (3.3%)	0 (0%)	58 (96.7%)	1 (1.7%)	1 (1.7%)

The use of non-official or blank paper for prescriptions was studied. This is quite a common practice in Sudan given the low resources setting. We needed to ensure that even blank papers included necessary information, such as the hospital name and date of the prescription. Official prescription paper writing was used in 73.3% of Cycle one prescriptions, improving to 91.7% in Cycle two, but slightly decreased to 81.7% in Cycle three. The hospital title was recorded in 73.3% of prescriptions in Cycle one, which improved to 95% in Cycle two and further got better with 96.7% in Cycle three. In the same way, documentation of the date was seen in 35% of prescriptions in Cycle one, which improved to 93.3% during Cycle two and 96.7% in Cycle three, as illustrated in Table 2.

**Table 2:** Use of official paper and inclusion of hospital title and date

Parameters	First cycle (n=60), n (%)	Second cycle (n=60), n (%)	Third cycle (n=60), n (%)
Use of official paper	44 (73.3%)	55 (91.7%)	49 (81.7%)
Inclusion of hospital title	44 (73.3%)	57 (95%)	58 (96.7%)
Inclusion of the date	21 (35%)	56 (93.3%)	58 (96.7%)

Patient information and diagnosis

Baseline prescriptions indicated great inadequacy in most vital data fields; for example, 86.7% (52/60) of the prescriptions lacked patients’ full names. Following interventions, the absence of names had reduced considerably declining to 13.3% (8/60) by Cycle three of the audit. Correspondingly, patient age was elaborated on merely 3.3% (2/60) of occasions and weight on 15% (9/60) of them, at baseline. By the end of Cycle three, the rates of reporting age and weight had remarkably increased to 86.7% (52/60) and 91.7% (55/60), respectively. Furthermore, the first set of audited prescriptions was found to be completely devoid of diagnosis information or indication for usage. This defect was being widely attended to through process improvements, and 96.7% (58) of prescriptions included this information by Cycle three of the audit (Table 3).

**Table 3:** Documentation of patient Information

Patient Information	First Cycle (n=60), n (%)		Second Cycle (n=60), n (%)		Third Cycle (n=60), n (%)	
	Not written	Written	Not written	Written	Not written	Written
Full Name	52 (86.7%)	8 (13.3%)	44 (73.3%)	16 (26.7%)	8 (13.3%)	52 (86.7%)
Age	58 (96.7%)	2 (3.3%)	21 (35%)	39 (65%)	8 (13.3%)	52 (86.7%)
Weight	51 (85%)	9 (15%)	12 (20%)	48 (80%)	5 (8.3%)	55 (91.7%)
Diagnosis (Rationale for prescription)	60 (100%)	0 (0%)	4 (6.7%)	56 (93.3%)	2 (3.3%)	58 (96.7%)

Prescribed drugs information

The audit demonstrated progressive improvement in the drug information prescription practice across the three cycles. The generic name was documented in all prescribed medications within the prescription in 88.3% (53/60) of the prescriptions by the third cycle, compared to 50% (30/60) in the first cycle. Drug concentration or strength was written in 28.3% (17/60) of the prescriptions in the first cycle, increasing to 66.7% (40/60) in the second cycle and 90% (54/60) in the third cycle. Partial documentation of drug strength decreased from 16.7% (10/60) in the first cycle to 6.7% (4/60) in the third cycle. Drug dose was omitted in 40% (24/60) of the first cycle prescriptions, declining to 0% (0/60) by the third cycle (Table 4).

**Table 4:** Documention of prescribed drugs information

Drug Information	First Cycle (n=60), n (%)			Second Cycle (n=60), n (%)			Third Cycle (n=60), n (%)		
	Written	Partially written	Not written	Written	Partially written	Not written	Written	Partially written	Not written
Generic Name	30 (50%)	15 (25%)	15 (25%)	42 (70%)	10 (16.7%)	8 (13..3%)	53 (88.3%)	4 (6.7%)	3 (5%)
Drug Strength	17 (28.3%)	10 (16.7%)	33 (55%)	40 (66.7%)	10 (16.7%)	10 (16.7%)	54 (90%)	4 (6.7%)	2 (3.3%)
Drug dose	32 (53.3%)	4 (6.7%)	24 (40%)	58 (96.7%)	1 (1.7%)	1 (1.7%)	59 (98.3%)	1 (1.7%)	0 (0%)
Drug Route	50 (83.3%)	5 (8.3%)	5 (8.3%)	58 (96.7%)	1 (1.7%)	1 (1.7%)	59 (98.3%)	1 (1.7%)	0 (0%)
Drug frequency	29 (48.3%)	3 (5%)	26 (46.7%)	57 (95%)	1 (1.7%)	2 (3.3%)	58 (96.7%)	1 (1.7%)	1 (1.7%)
Drug Duration	17 (28.3%)	7 (11.7%)	36 (60%)	47 (78.3%)	3 (5%)	10 (16.7%)	52 (86.7%)	5 (8.3%)	3 (5%)

In the initial cycle, 46.7% (28/60) of the prescriptions did not contain the drug frequency of administration, while 96.7% (58/60) of the third-cycle prescriptions sample contained written drug frequency of administration. Recording of medication route of administration improved from 83.3% (50/60) in the first cycle to 96.7% (58/60) in the second cycle, with this percentage increasing to 98.3% (59/60) in the third cycle. Substantial improvement was evident in the documentation of drug duration, with recording practice increasing from 28.3% (17/60) in the first cycle to 86.7% (52/60) in the third cycle. Partial or missing documentation dropped from 71.7% (43/60) to 13.3% (8/60) over the same period (Table 4). Non-standard abbreviations were only used in 1.7% (1/60) in the first cycle, and no documented use of non-standard abbreviations was noted thereafter.

Prescriber information

In the first cycle, it was observed that 96.7% of prescribers failed to include their names, and 65% did not provide a signature. Following an intervention, the third cycle demonstrated significant improvement, with 81.7% of prescribers including their names and 93.3% providing signatures (Table 5).

**Table 5:** Documentation of prescriber’s information

Prescriber’s Information	First Cycle (n=60), n (%)		Second Cycle (n=60), n (%)		Third Cycle (n=60), n (%)	
	Written	Not Written	Written	Not Written	Written	Not Written
Prescriber Name	2 (3.3%)	58 (96.7%)	24 (40%)	36 (60%)	49 (81.7%)	11 (18.3%)
Prescriber Signature	21 (35%)	39 (65%)	46 (76.7%)	4 (23.3%)	56 (93.3%)	4 (6.7%)

## Discussion

This was a prescription audit carried out in a hospital in Khartoum, Sudan, over three cycles. We found the prescription date missing on 3.3% of prescriptions. This was substantially better than the 88.7% reported in a Khartoum study and the 31% in a Saudi Arabian tertiary hospital study [7,11]. Prescription eligibility reached 96.7% in our audit, an improvement from the first cycle and higher than the 88.2% reported in Khartoum [7], 87% in Pakistan [12], and 68% in India [13]. Improved prescription eligibility increases patient safety, reduces misinterpretation, and improves communication among healthcare providers [12-15]. Prescriber names and signatures appeared on 81.7% and 93.3% of prescriptions, respectively, vital for verifying prescription authenticity. These results show considerable improvement over a Khartoum study, which found prescriber names and signatures on only 60.7% and 29.6% of prescriptions, respectively [7]. 

Significant shortcomings in prescription completeness were found during this audit. The patient’s full name, which consists of three names, was missing from a significant majority of prescriptions (86.7%). This result is consistent with a study that found a similar insufficiency in 81.2% of prescriptions at Ribat University in Sudan [6]. On the other hand, although it is uncertain if this included all three names, a previous study conducted in Khartoum primary health care centers, Sudan, showed complete patient name inclusion on all prescriptions [7]. Writing the patient’s full name will prevent medication errors by ensuring the correct medication is dispensed to the right patient. 

Patient weight was missing in 85% of cases, and patient age was missing in 96.7% of cases, further decreasing the quality of prescriptions. These exclusions are similar to the study conducted at Ribat University Hospital, which found that 93.5% of prescriptions lacked patient age [6]. Although the study conducted by the Khartoum PHC centers revealed a decreased rate of missing age (61.7%), it also discovered that 95% of prescriptions did not include the patient’s weight [7]. These findings highlight a systematic inability to document vital patient data required for determining the appropriate dosage of medication, especially for children. 

It is concerning that not a single prescription in this audit included a diagnosis or rationale for the drug’s use at baseline. This is similar to the study conducted at Ribat University Hospital, which found that 94% of prescriptions did not include the diagnosis [6]. However, the Khartoum PHC centers study showed somewhat better performance, with half of prescriptions providing a diagnosis [7]. Patient safety and the standard of care are seriously jeopardized when diagnostic data are lacking. This information helps the pharmacist understand the purpose of the medication and identify any potential drug interactions or contraindications. 

Initially, only 50% of prescriptions utilized generic drug names. This aligns with findings from a study in primary healthcare centers in Khartoum, Sudan (where 49.8% used generic names for prescribed medication), but falls short of best practices [7]. Notably, a study at Ribat University Hospital, Khartoum, reported a significantly lower rate of generic prescribing (19.5%) [6]. By the end of our audit, 88.3% of the prescriptions used generic drug names. Prescribing generics empowers patients with cost-conscious medication choices by allowing pharmacists to offer more affordable yet equally effective alternatives. 

Drug strength was omitted in 55% of the initially studied prescriptions, considerably higher than the 3.2% reported in a study from Jizan [8]. This finding is supported by the Ribat University Hospital study, which observed a 36% omission rate [6]. Our final results revealed that 90% of the prescriptions included the drug concentration. Inaccurate strength information poses a significant risk of medication errors due to the critical role of concentration in dosage calculations. Essential information omissions included dose omission with only 53.3% of prescriptions initially including drug dosage, significantly higher than the 5% omission rate seen in the Jizan study [8] and exceeding the 30.6% omission rate observed in Khartoum [7]. In our audit, we reduced this to zero omissions by the third cycle.

Drug frequency was documented in 48.3% of prescriptions at the beginning of the audit, which improved to 96.7% by the end. Additionally, drug route was documented in 96.7% of prescriptions. Initially, treatment duration was documented in only 28.3% of prescriptions, higher than the 34.8% and 25.7% omission rates reported in the Jizan and Ribat University Hospital studies, respectively [6,9]. This was improved to 86.7% by the end of our audit.

Recommendations

Education and Training

The importance of education was clearly evident through this audit. The authors recommend conducting regular educational programs for prescribers to raise awareness of the importance of accurate and complete prescription information and the potential consequences of data omissions.

Enhancing Generic Prescribing

The authors recommend the implementation of strategies to encourage and promote the use of generic drug names in prescribing practices, such as setting a local pharmacy alert system to prevent the dispensing of prescriptions containing trade names unless the necessity of such a prescription is explained.

Improving Data Completeness

The importance of documenting drug strength, dosage, frequency, route, and treatment duration on all prescriptions should be emphasized.

Limitations

This audit was conducted in the outpatient clinic of a pediatric department, which is a single department. This may limit the generalizability of the findings. This limitation could be addressed in future audits by expanding the audit scope to other departments and multiple hospitals across the state or at a national level. Additionally, while the educational intervention improved prescribing practices, its impact was assessed over a relatively short period. A longer follow-up may be necessary to evaluate the sustainability of these improvements. However, this was challenging for the team due to the rotational nature of their roles. Although the intervention included interactive training, feedback forms, and reinforcement through posters and social media, engagement levels among participants were not formally assessed. Variability in individual participation and adherence to recommended prescribing standards may have influenced the overall effectiveness of the intervention. Finally, external factors such as workload pressures and staffing changes may have affected prescribing practices independently of the audit. Future audit cycles could benefit from a more comprehensive evaluation of these factors to enhance the relevance of the findings.

## Conclusions

The results highlight the critical benefits of systematic documentation practices in contributing to prescription accuracy and mitigating clinical errors. Structured initiatives improved markers of prescription eligibility and authentication, thereby strengthening accountability and interprofessional collaboration. These advances conform to the international standards for safety, showing that standard protocols reduce iatrogenic hazards while simultaneously ensuring patient safety and optimizing therapeutic outcomes. Clarity and completeness in prescription writing emerges, therefore, as one of the most important strategies for preventing misinterpretation and fostering confidence in health delivery systems.

After interventions were carried out, documentation of prescriptions improved dramatically. Missing details, including patient name and age, weight, and diagnosis, were successfully taken care of. There is a need for further work in this area. While developed countries have electronic systems to mitigate the problem of missing data, local adaptations within low-resource settings such as ours and other constraints would be required to bridge such gaps and thus enhance patient safety. The findings indicate that substantial deficiencies in the quality of prescribing information, in particular, regarding drug strength, treatment duration, and frequency, increase the risk for medication errors and suboptimal patient outcomes.
